# Magnesium Sulfate Combination Therapy for Aconitine‐Induced Electrical Storm

**DOI:** 10.1111/anec.70124

**Published:** 2025-10-30

**Authors:** Liujiang Ran, Jun Si, Yanyan Liu

**Affiliations:** ^1^ Department of Pre‐hospital Emergency Chongqing University Central Hospital (Chongqing Emergency Medical Center) Chongqing China; ^2^ Department of Infectious Disease of Jiangbei Campus The First Affiliated Hospital of Army Medical University Chongqing China

**Keywords:** aconitine, electrical storm, hemoperfusion, magnesium sulfate, multimodal intervention

## Abstract

We present the case of a 74‐year‐old man who developed 28 episodes of electrical storm secondary to aconitine poisoning from homemade herbal wine consumption, followed by complete recovery through 28 electrical cardioversions, hemopurification, and continuous magnesium sulfate infusion after 4 days. This case suggests that magnesium sulfate, by antagonizing calcium overload and stabilizing membrane potential, forms a synergistic effect with electrical resuscitation and hemopurification, and is the key to reversing the electrical storms caused by aconitine poisoning.

## Graphical abstract

Treatment course of 28 episodes of electrical storm caused by aconitine poisoning with combined magnesium sulfate therapy.

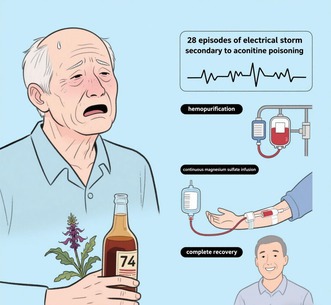

## 1 Introduction

Aconitum plant poisoning predominantly affects middle‐aged and elderly men, with refractory ventricular fibrillation (VF) being the principal cause of mortality (Chan 2009). This critical emergency frequently encountered in emergency departments typically originates from accidental ingestion of herbal wines containing aconitine‐derived botanicals (e.g., processed aconite root, wild Aconitum) (Chan 2009; Zhou et al. 2021; Wang et al. 2023). The aconitine induces lethal arrhythmias through persistent activation of sodium channels, with a 10%–30% mortality rate (Friese et al. 1997). Despite increasing incidents of homemade herbal wine consumption, limited literature exists regarding geriatric patients presenting with sustained malignant arrhythmias requiring multimodal interventions. This report integrates a prototypical case with contemporary evidence to delineate optimized management strategies for aconitine cardiotoxicity.

## 2 Case Report

A 74‐year‐old male patient was transferred to the emergency department of our hospital at 02:21 on March 2, 2025, due to sudden onset of profuse sweating for 5+ h after consuming herbal wine. According to the family, the patient developed excessive sweating, restlessness, and agitation approximately 5 h earlier after drinking approximately 100 mL of self‐prepared herbal wine, with no limb convulsions or impaired consciousness. The patient was initially diagnosed with aconitine poisoning at a local hospital. An electrocardiogram (ECG) revealed supraventricular tachycardia. Following intravenous administration of verapamil and 100 J electrical cardioversion, the heart rate was temporarily controlled at around 150 beats per minute (bpm). For further treatment, the family contacted emergency services (via 120) to transfer the patient to our emergency department.

**FIGURE 1:**
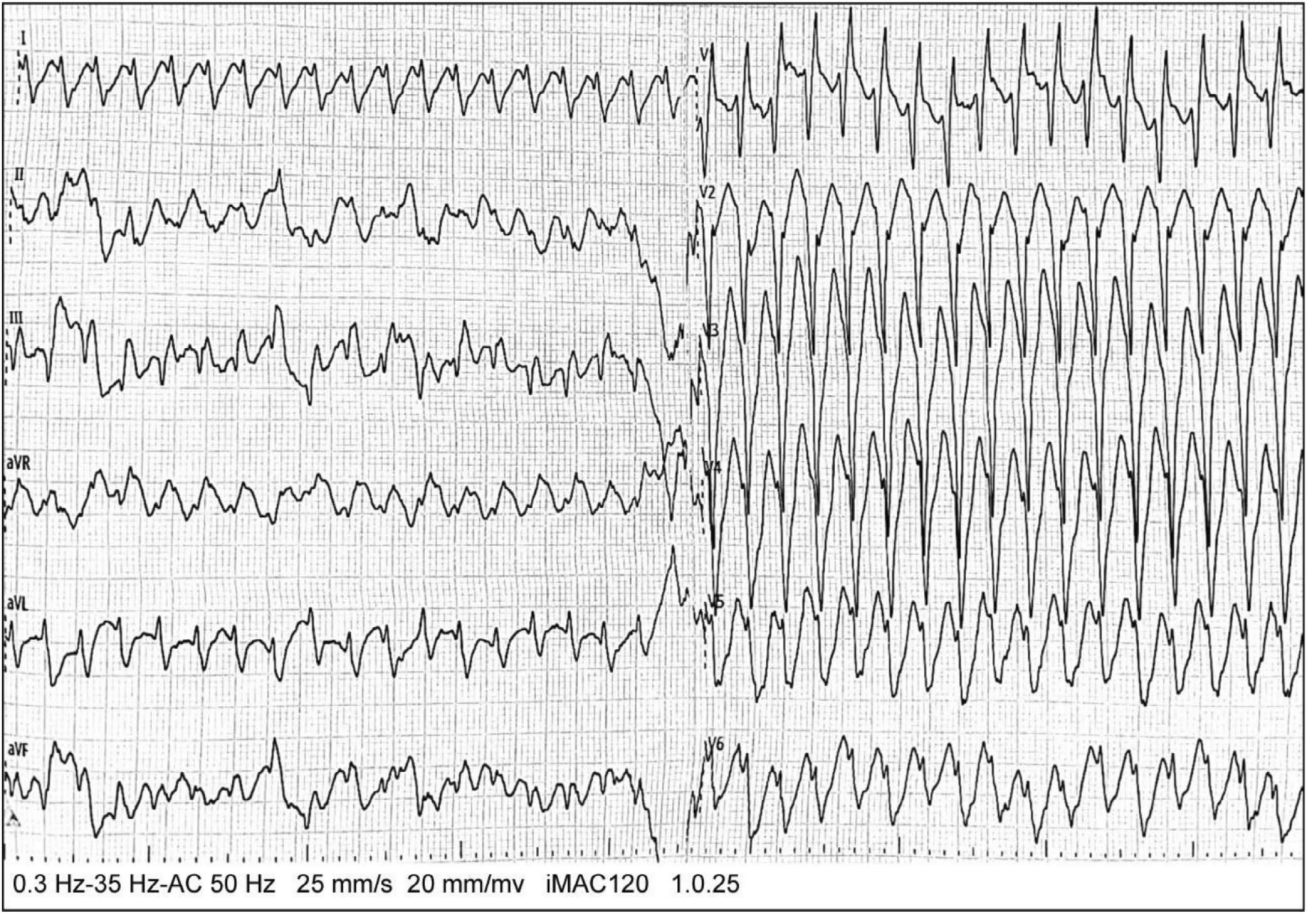
ECG on admission. Ventricular rate of 246 bpm with regular rhythm, absence of discernible sinus P‐waves, and widened QRS complexes (144 ms duration) demonstrating abnormal morphology and R‐wave predominance in lead aVR, diagnostic of VT.

The patient was previously healthy. On admission, physical examination showed: body temperature 36.5°C, pulse 250 bpm, respiratory rate 33 breaths/min, blood pressure 76/32 mmHg. The patient exhibited a distressed appearance and lethargic consciousness, with a Glasgow Coma Scale (GCS) score of 8. Immediate arterial blood gas analysis revealed: pH 7.28, pCO_2_ 22 mmHg, pO_2_ 170 mmHg, lactate (Lac) 11.0 mmol/L, and base excess (BE) – 14.2 mmol/L. Cardiac enzyme tests showed: CK‐MB 6.04 ng/mL, Myoglobin (Myo) 110 ng/mL, and cardiac troponin I (cTnI) 0.018 ng/mL. Electrolyte levels were within normal ranges. Continuous cardiac monitoring indicated sustained ventricular tachycardia (VT) (Figure 1).

**FIGURE 2:**
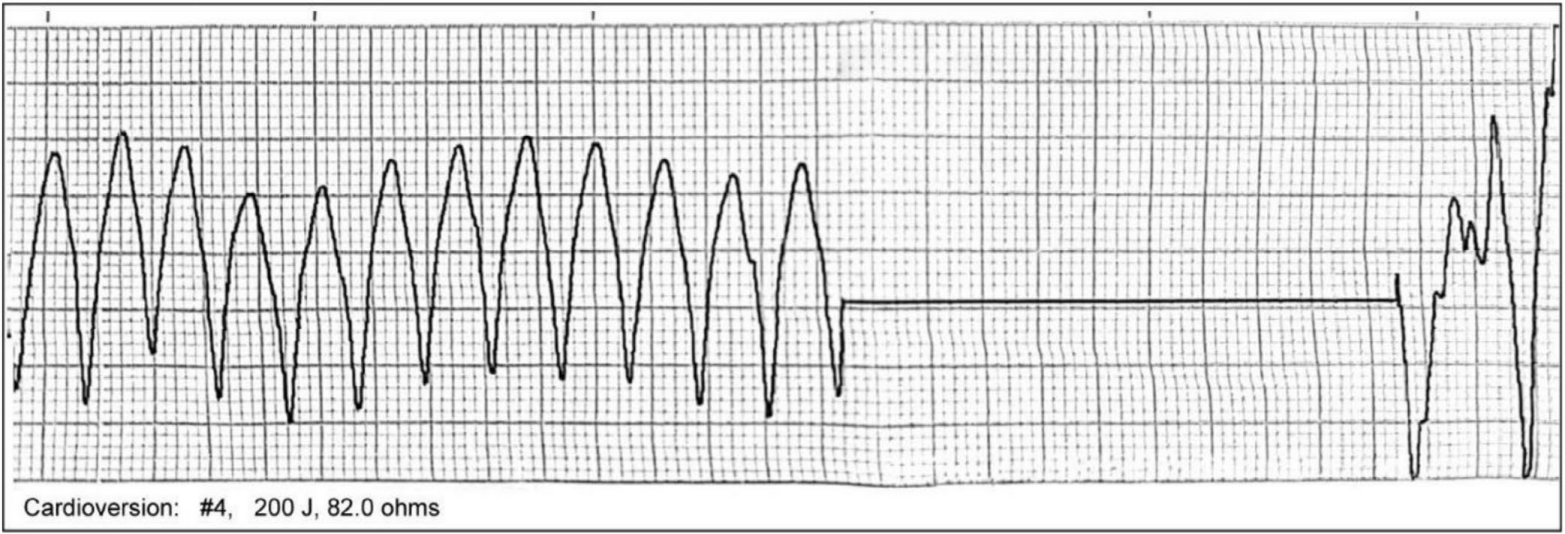
During cardioversion. Absence of identifiable normal QRS‐T complexes with a ventricular rate of 250 beats per minute (bpm), diagnostic of VT.

Following consultations with the cardiology and ICU departments, the patient was immediately administered one synchronized biphasic electrical defibrillation at 100 J. Post‐defibrillation, cardiac monitoring continued to show VF (Figure 2). A subsequent synchronized electrical cardioversion at 150 J was performed once, but no improvement was observed. This was followed by 10 consecutive attempts of synchronized (200 J) and asynchronized cardioversion, along with an intravenous bolus of lidocaine 100 mg. Despite these interventions, the patient experienced intermittent episodes of VT. Subsequently, the patient's oxygen saturation progressively declined, prompting immediate endotracheal intubation. Repeat arterial blood gas analysis revealed: pH 7.29, pCO_2_ 38 mmHg, pO_2_ 136 mmHg, Lac 6.4 mmol/L, and base excess (BE) −7.7 mmol/L. After discussion with the family, the patient was admitted for inpatient care. During preparation for ICU transfer, the patient experienced recurrent arrhythmia, with a heart rate reaching 250 bpm. Eight additional attempts of synchronized and asynchronized cardioversion at 200 J were performed, resulting in a heart rate reduction to 184 bpm and blood pressure stabilization at 103/79 mmHg. However, cardiac monitoring continued to indicate frequent episodes of VT. The patient was subsequently transferred to the inpatient ward at 03:54.

**FIGURE 3:**
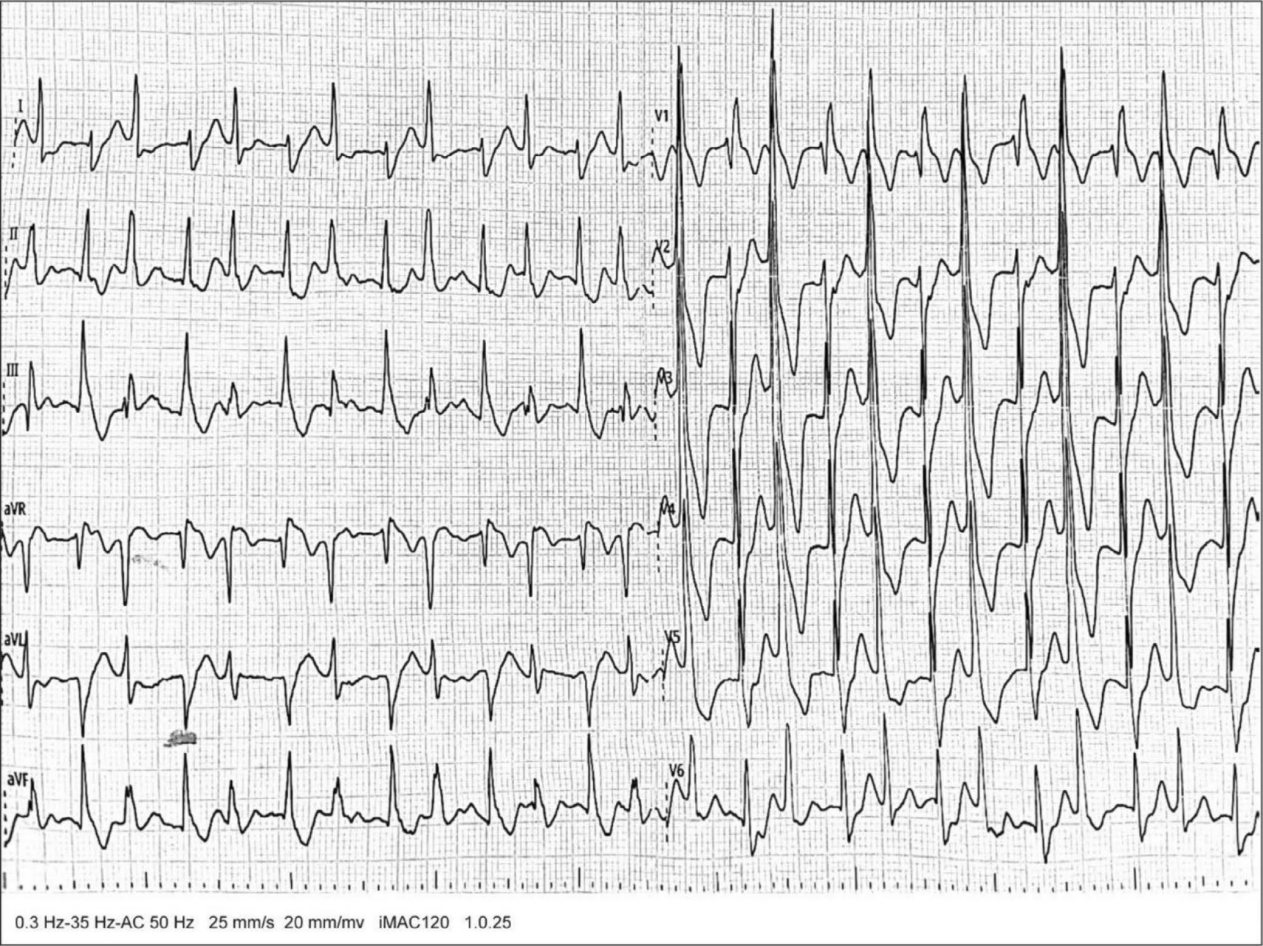
Typical VT. Ventricular rate of 153 bpm. No apparent sinus P waves in lead I. Wide and deformed QRS complexes with a duration of 118 ms, exhibiting rS and Rs morphologies alternating. Alternating R'‐R' intervals of varying lengths, suggestive of bidirectional VT.

**FIGURE 4:**
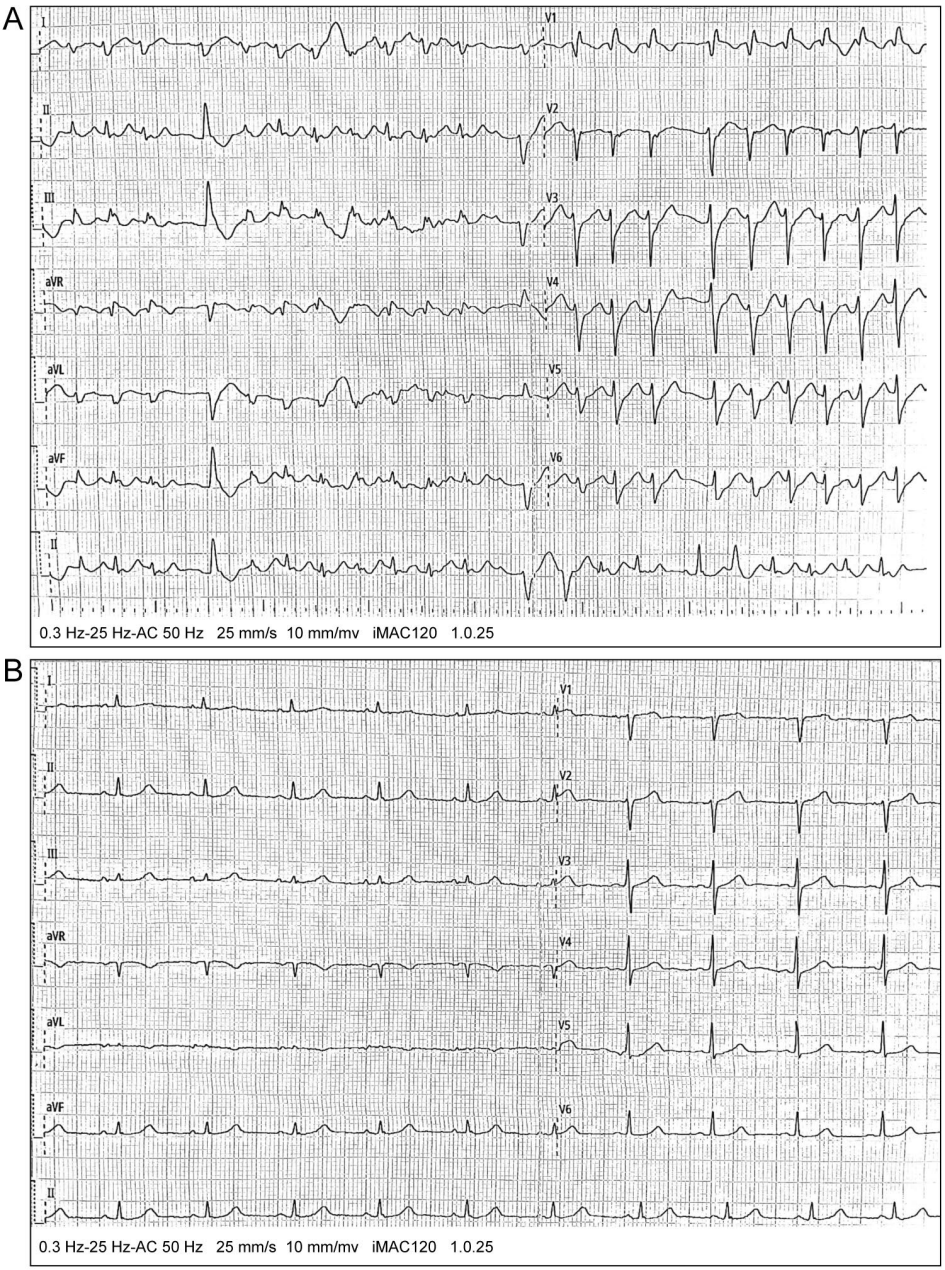
Post‐treatment and conversion to sinus rhythm. (A) After cardioversion and magnesium sulfate treatment, the ventricular rate has decreased. However, the QRS complexes are widened with abnormal morphology, accompanied by irregular R'‐R' intervals and polymorphic QRS waveforms, suggesting polymorphic VT. (B) Sinus rhythm with a ventricular rate of 75 bpm.

After transfer to the inpatient ward, cardiac monitoring continued to reveal frequent ventricular arrhythmias. Immediate interventions included lidocaine and esmolol for heart rate control, ice cap application for cerebral protection, fluid resuscitation, cathartics, and aggressive hemoperfusion therapy. Despite these measures, the patient continued to experience frequent VT (Figure 3), prompting 8 additional asynchronized biphasic defibrillations at 200 J. Repeat blood gas analysis during this period showed: pH 7.45, pCO_2_ 35 mmHg, pO_2_ 154 mmHg, Lac 2.0 mmol/L, ionized calcium 0.96 mmol/L, potassium 3.3 mmol/L, sodium 141 mmol/L. Based on these results, 3 g magnesium sulfate was administered intravenously, followed by a continuous infusion of 1–2 g/h magnesium sulfate via syringe pump, which significantly reduced the ventricular rate (Figure 4A). By the next day, sinus rhythm was restored with a ventricular rate of 75 bpm (Figure 4B).

Subsequent Treatment: Hemoperfusion + continuous renal replacement therapy (CRRT), sedation and analgesia, vasopressor support, maintenance of internal homeostasis, antibiotics and gastric protection, the patient's condition improved over 4 days, and prior to discharge, the patient's ECG, arterial blood gas analysis, cardiac enzyme panel, and myocardial injury markers were all within normal ranges. The treatment protocol is summarized in Table 1.

**TABLE 1:** Key time points in the treatment of aconitine‐induced electrical storm.

Time	Event	Progress	Intervention	Effect
2025.03.01 23:21	Local Hospital Visit	Supraventricular Tachycardia	Electrical cardioversion (1), intravenous verapamil	Conversion to sinus rhythm, heart rate decreased to 150 bpm
2025.03.02 02:21	Our Emergency Department	Sustained VF, Intermittent VT	Electrical cardioversion (20), lidocaine, endotracheal intubation	Recurring VT, heart rate 184 bpm
2025.03.02 03:54	ICU	Frequent VT/VF	Lidocaine, esmolol, electrical cardioversion (8), magnesium sulfate, HP + CRRT	Ventricular rate decreased to 160 bpm
2025.03.03 10:57	ICU	Return to Sinus Rhythm		Ventricular rate 75 bpm
2025.03.06 15:30	Discharge	Sinus Rhythm	—	Sinus rhythm 69 bpm

## 3 Discussion

Aconitine poisoning induces cardiac arrhythmias of varying severity in most patients, with ventricular arrhythmias and conduction blocks being the most common and most likely to cause death. The mechanism is related to its direct action on ventricular myocardium, opening Na^+^ channels in ventricular myocytes, accelerating Na^+^ influx, causing asynchronous repolarization of adjacent ventricular myocardium and facilitating re‐entry, leading to VT and VF, as well as excitation of cardiac vagus nerves that reduces sinoatrial node automaticity and conductivity, resulting in sinus bradycardia (Xiao et al. 2011). In this case, the patient's advanced age combined with severe acidosis further exacerbated myocardial electrophysiological instability, manifesting as refractory electrical storm (28 electrical cardioversions), which is highly consistent with the characteristics of fatal arrhythmias reported in previous literature.

The principles of managing aconitine poisoning include gastric lavage, catharsis, diuresis, fluid resuscitation, stabilization of vital signs, and early hemoperfusion, with the key being effective control of life‐threatening arrhythmias (Coulson et al. 2017). In this case, the patient exhibited persistent malignant arrhythmias without significant improvement despite repeated electrical cardioversion and combined antiarrhythmic drug therapy. Ultimately, multimodal interventions including hemoperfusion + CRRT and magnesium sulfate for electrolyte balance successfully reversed the condition. The key aspects of this rescue included three areas: (1) Combined use of electrical cardioversion and antiarrhythmic drugs: Electrical cardioversion is the first‐line treatment for hemodynamically unstable arrhythmias (Markman and Nazarian 2019). However, for this persistent malignant arrhythmia, although electrical cardioversion temporarily restored sinus rhythm, the sustained sodium channel activation effect of aconitine led to recurrent arrhythmias, indicating the limitations of electrical intervention alone. This was confirmed in this case by the need for 28 consecutive defibrillations despite combined therapy with lidocaine (a sodium channel blocker) and esmolol (a β‐blocker). Administering 28 consecutive electrical cardioversions within 2 h posed a dual challenge for both the patient's tolerance and the physician's confidence. In this case, the absence of significant myocardial damage before and after cardioversion and at discharge, as indicated by cardiac enzyme and myocardial injury markers, fully demonstrated the safety of biphasic defibrillation, laying a foundation for future clinical rescues. (2) Core role of blood purification: CRRT corrects internal environmental disturbances and removes inflammatory mediators, particularly in patients with multi‐organ dysfunction. Early hemoperfusion effectively clears aconitine from the bloodstream (Ke et al. 2024), with literature recommending initiation within 6 h of poisoning for significant patient benefit. In this case, CRRT reduced Lac from 11.0 to 0.9 mmol/L, successfully reversing tissue hypoperfusion. (3) Metabolic and electrolyte management: Acidosis (pH < 7.3) and electrolyte imbalances can exacerbate arrhythmias and require dynamic monitoring and correction. In this case, ionized calcium decreased to 0.96 mmol/L during treatment, and a hypocalcemic environment may enhance aconitine toxicity. Magnesium sulfate stabilizes myocardial cell membrane potential by replenishing magnesium ions and corrects hypomagnesemia through synergistic effects, indirectly regulating calcium homeostasis and improving myocardial electrical stability (Kolte et al. 2014; Chen et al. 1988). According to the Expert Consensus on Diagnosis and Treatment of Acute Aconitum Alkaloid Poisoning, magnesium sulfate is classified as a Class IIa recommended drug for refractory ventricular arrhythmias (Evidence Level B) (Emergency Physician Branch of Chinese Medical Doctor Association et al. 2022). A study of 36 severe aconitine poisoning cases showed that early magnesium sulfate administration (≤ 6 h) increased the success rate of VF cardioversion by 40%. In this case, after 28 electrical cardioversions with persistent VT, the addition of magnesium sulfate successfully reduced the heart rate from 250 to 136 bpm. This effect aligns with literature reporting that magnesium agents reduce QT interval dispersion and lower the risk of reperfusion arrhythmias (Parikka et al. 1999). Additionally, the combined use of lidocaine in this case—lidocaine reduces abnormal impulses by blocking sodium channels, while magnesium sulfate inhibits calcium influx—synergistically antagonized aconitine toxicity at different ion channel levels. A study demonstrated that combining magnesium sulfate with lidocaine significantly shortens the duration of arrhythmia and hospitalization in patients with aconitine‐induced arrhythmias (Coulson et al. 2017).

There were certain shortcomings in the management of this patient: Gastric lavage and emesis were withheld due to concerns that vagal nerve stimulation from these procedures could exacerbate arrhythmias (Shang et al. 2024), as the patient presented with malignant arrhythmias upon admission. Serum magnesium levels were not dynamically monitored, making it impossible to confirm hypomagnesemia. Some scholars argue that high‐dose magnesium sulfate in the absence of hypomagnesemia may induce hypotension or muscle weakness, necessitating cautious titration. Further randomized controlled trials are required to clarify the dose–response relationship of magnesium sulfate at different poisoning stages and its safety when combined with other drugs (e.g., β‐blockers). Future efforts should standardize the timing and dosage of magnesium sulfate administration and strengthen serum magnesium monitoring to optimize treatment strategies for severe aconitine poisoning.

## 4 Conclusion

For patients suspected of aconitine poisoning, early identification of arrhythmia types and timely intervention are critical. This case demonstrates that in the management of aconitine‐induced cardiac electrical storm, the synergistic interplay of magnesium sulfate, electrical cardioversion, and blood purification is pivotal to successful resuscitation. For such patients, early combination therapy with magnesium sulfate alongside electrical cardioversion is recommended. Additionally, public education should be strengthened to prevent the consumption of self‐prepared herbal wines containing aconitine.

## Author Contributions

**Liujiang Ran** and **Jun Si:** investigation, analysis and writing. **Yanyan Liu:** investigation, review and editing.

## Conflicts of Interest

The authors declare no conflicts of interest.

## Data Availability

The data that support the findings of this study are available from the corresponding author upon reasonable request.
